# Triplet–triplet annihilation-based photon-upconversion to broaden the wavelength spectrum for photobiocatalysis

**DOI:** 10.1038/s41598-022-13406-8

**Published:** 2022-06-07

**Authors:** Se-Yeun Hwang, Dayoon Song, Eun-Ji Seo, Frank Hollmann, Youngmin You, Jin-Byung Park

**Affiliations:** 1 Department of Food Science and Biotechnology, Ewha Womans University, Seoul, 03760 Republic of Korea; 2 Division of Chemical Engineering and Materials Science, and Graduate Program in System Health Science and Engineering, Ewha Womans University, Seoul, 03760 Republic of Korea; 3 Department of Biotechnology, Delft University of Technology, Van der Maasweg 9, 2629 HZ Delft, The Netherlands

**Keywords:** Nanoscale materials, Enzymes

## Abstract

Photobiocatalysis is a growing field of biocatalysis. Especially light-driven enzyme catalysis has contributed significantly to expanding the scope of synthetic organic chemistry. However, photoenzymes usually utilise a rather narrow wavelength range of visible (sun)light. Triplet–triplet annihilation-based upconversion (TTA-UC) of long wavelength light to shorter wavelength light may broaden the wavelength range. To demonstrate the feasibility of light upconversion we prepared TTA-UC poly(styrene) (PS) nanoparticles doped with platinum(II) octaethylporphyrin (PtOEP) photosensitizer and 9,10-diphenylanthracene (DPA) annihilator (PtOEP:DPA@PS) for application in aqueous solutions. Photoexcitation of PtOEP:DPA@PS nanoparticles with 550 nm light led to upconverted emission of DPA 418 nm. The TTA-UC emission could photoactivate flavin-dependent photodecarboxylases with a high energy transfer efficiency. This allowed the photodecarboxylase from *Chlorella variabilis* NC64A to catalyse the decarboxylation of fatty acids into long chain secondary alcohols under green light (*λ* = 550 nm).

## Introduction

Photobiocatalysis is a rapidly growing field of biocatalysis^1–5^. Phototrophic microorganisms^6,7^, light-induced enzyme reactions^8–15^, and light-driven cofactor regeneration systems^16,17^ have contributed to expanding the toolbox of biocatalysis for organic synthesis. Light-driven transformations have opened up new avenues for the environmentally benign synthesis of chemicals^1–5,8–10,15,18–20^ and fuels^21–23^.

Envisioning solar power to fuel the promising approaches mentioned above, however, is limited by the generally narrow use of the photon energy provided by sunlight. For example, reactions using flavin photocatalysts efficiently utilise wavelengths between 300 and 500 nm thereby leaving a significant part of solar energy unused.

Light upconversion (UC) possibly solves this issue. UC comprises the generation of higher-energy photons from low-energy photons (Figure S1)^24–28^. Upconverted light can be generated by combining two-photon absorption dyes, nanoparticles doped with rare-earth elements and triplet–triplet annihilation-based UC (TTA-UC) materials^29^. Among the systems, TTA-UC has been used most extensively, because the light upconversion could be achieved with non-coherent and low-power photons, to a rather high quantum efficiency (Φ_UC_) of 1–5%^30–32^. TTA-UC has been used for photocatalysis^30,33–35^, solar energy harvesting^36^, drug delivery and activation^37^, and luminescence bioimaging^38–40^.

Established TTA-UC systems however are not compatible with aqueous reaction conditions^41^, which can be solved by incorporation of the TTA-UC components into the inner space within water-stable materials^27,42^. This may allow to enhance the quantum efficiency (Φ_UC_) because both triplet–triplet energy transfer and TTA can be accelerated via close contacts among the TTA-UC components (Figure S2).

In this study, we have prepared TTA-UC poly(styrene) (PS) nanoparticles which were doped with a platinum(II) octaethylporphyrin (PtOEP) photosensitiser and the 9,10-diphenylanthracene (DPA) annihilator (PtOEP:DPA@PS) for application in aqueous reaction systems (Figure S2). Photoexcitation of PtOEP:DPA@PS nanoparticles with 550 nm light lead to an upconverted emission of DPA at 418 nm. Hence, an unproductive wavelength for e.g. flavin excitation can be upconverted to a wavelength lying in the productive wavelength range.

To test our hypothesis, we chose the light-activated fatty acid decarboxylase from *Chlorella variabilis* NC64A (*Cv*FAP)^18,43^. *Cv*FAP utilises a flavin cofactor, which in its photactivated form initiates the decarboxylation of carboxylic acids. As established previously, *Cv*FAP can productively use wavelengths between 300 and 500 nm. Green light barely promotes *Cv*FAP-catalysis. *Cv*FAP and its mutants catalyse the irreversible decarboxylation of saturated and unsaturated fatty acids but also hydroxy fatty acids^15,18^, amino fatty acids, and ester bond-containing fatty acids (e.g., (*Z*)-11-(heptanoyloxy)undec-9-enoic acid)^19^, generating long chain secondary alcohols, long chain aliphatic amines and esters, respectively. Engineered variants of *Cv*FAP have been reported for the conversion of short-chain carboxylic acids^22^ as well as for the kinetic resolution of some α-substituted carboxylic acids and unnatural amino acid phosphinothricin^11,44^.

**Figure 1:**
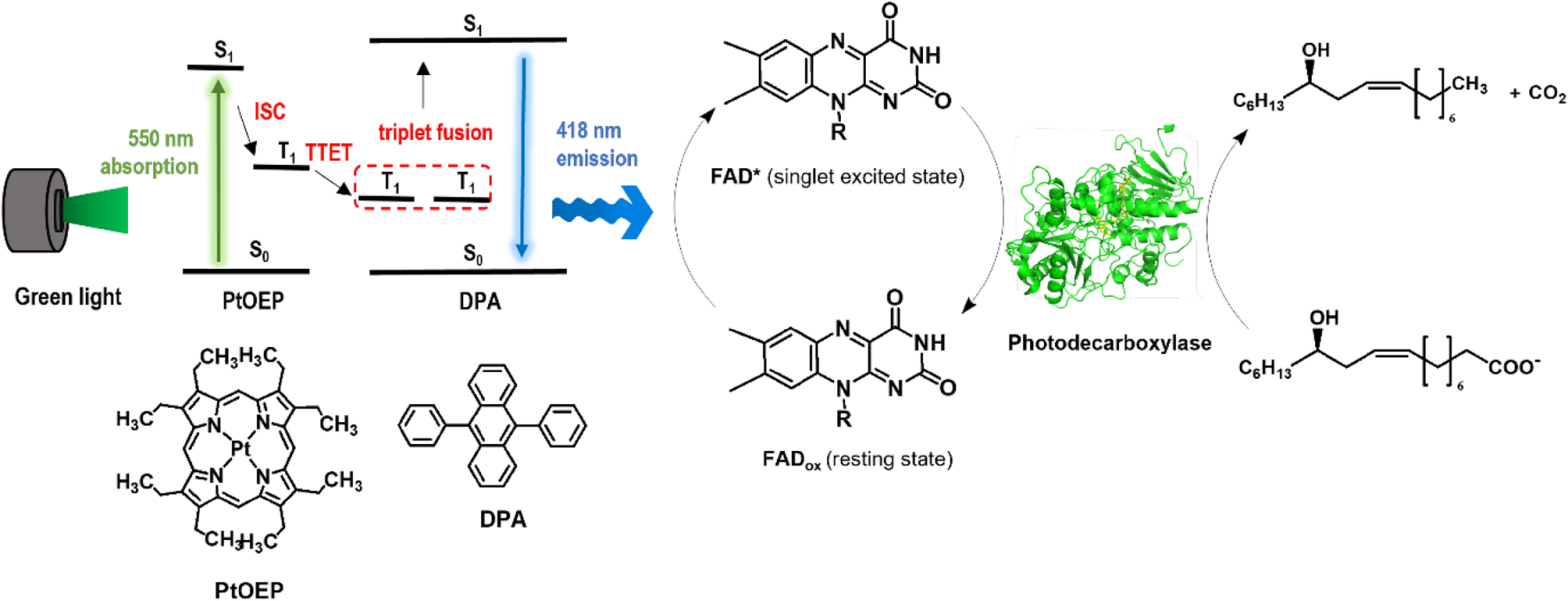
Overall concept of triplet–triplet annihilation-based photon-upconversion (TTA-UC) for light-driven enzyme catalysis. The TTA-UC allows conversion of long wavelength (*λ* = 550 nm) to short wavelength light (*λ* = 418 nm), which activates FAD in the enzymes for catalysis (see the Figures S1 and S2 for details). PtOEP: platinum(II) octaethylporphyrin, DPA: 9,10-diphenylanthracene, ISC: intersystem crossing, TTET: triplet–triplet energy transfer.

Overall, we envision UP to enlarge the wavelength scope of photo(bio)catalytic transformations such as the *Cv*FAP-catalysed carboxylic acid decarboxylation (Fig. 1).

## Results and discussion

### Light upconversion by PS nanoparticles

TTA-UC nanoparticles were prepared by flash nanoprecipitation of 1.0 mL THF containing 1.0 wt% PS, 0.01 wt% PtOEP and 0.2 wt% DPA in stirred 9.0 mL milli-Q^45^. The PS nanoparticles were purified by repeated centrifugation and decantation of the supernatant. The prepared nanoparticles were spherical with an average diameter of 275 nm (Figure S3). Efficiencies for encapsulation of PtOEP and DPA in PS were determined by UV–vis absorption spectroscopy to be 29% and 24%, respectively (Figure S4 and Methods). These values correspond to molar concentrations of 3.5 μM (0.53 wt% relative to polymer) and 130 μM (8.9 wt% relative to polymer) for PtOEP and DPA, respectively. The TTA-UC nanoparticle suspension was stable for several days in air-equilibrated milli-Q water.

**Figure 2:**
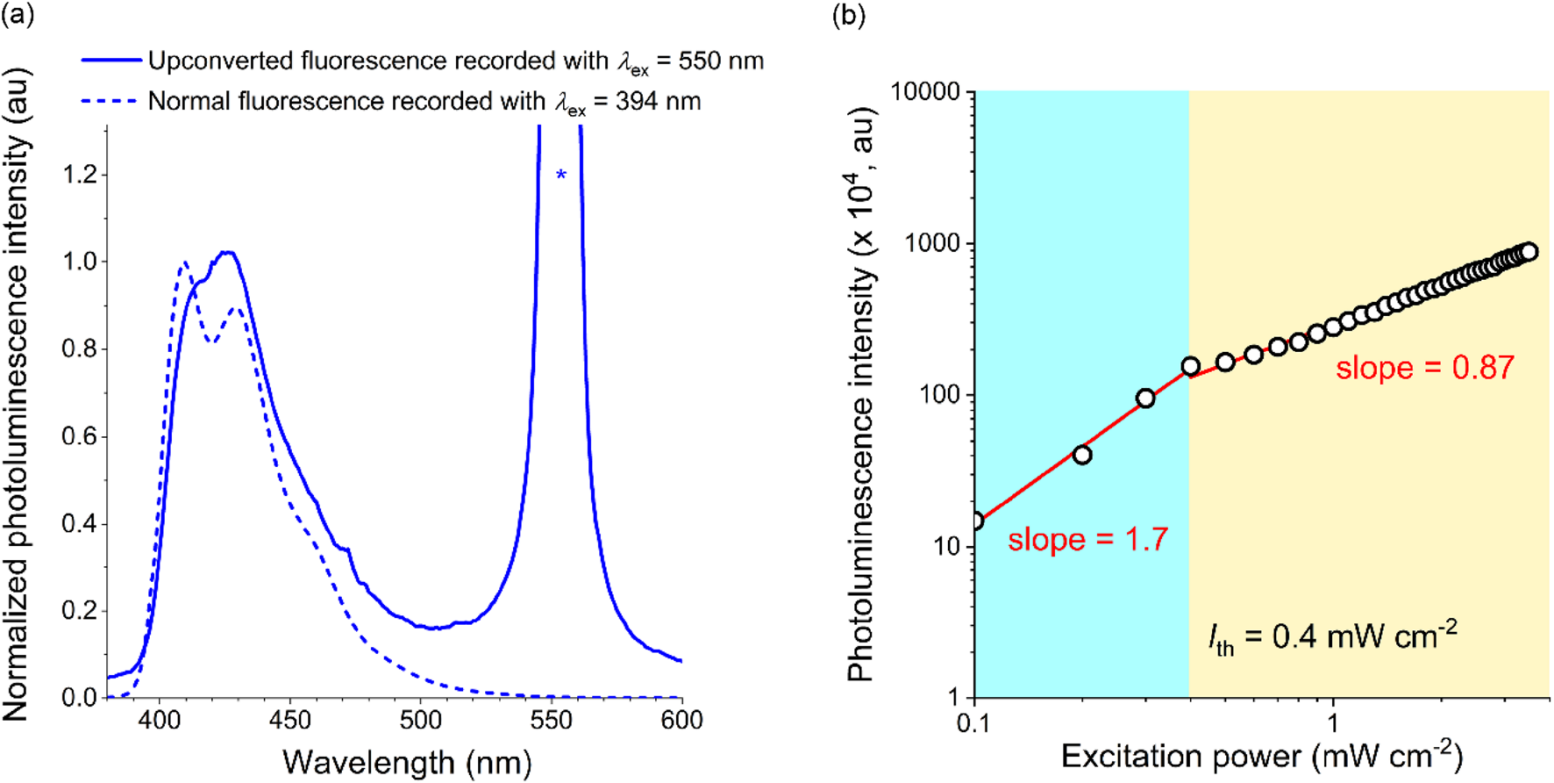
Upconverted fluorescence emission from TTA-UC nanoparticles. (**a**) Photoluminescence spectra of the TTA-UC nanoparticle (i.e., PtOEP:DPA@PS nanoparticle) suspension (milli-Q water) recorded upon the photoexcitation of PtOEP at a wavelength of 550 nm (solid line) and the direct photoexcitation of DPA at a wavelength of 394 nm (dashed line). The peak marked with an asterisk is the 550 nm excitation beam. (**b**) A double-logarithmic plot of the photoluminescence intensity as a function of the excitation power. The sky-blue and the yellow regions indicate upconverted fluorescence where the bimolecular TTA and the unimolecular radiative decay of DPA dominate.

The PtOEP:DPA@PS nanoparticles exhibited blue emission with a peak wavelength of 418 nm, upon photoexcitation of PtOEP at a wavelength of 550 nm (Fig. 2a). The 418 nm emission was from DPA, because an identical emission spectrum was observed under direct photoexcitation of DPA at a wavelength of 394 nm. Note that 10 μM DPA (THF) did not produce fluorescence emission upon direct excitation at a wavelength of 550 nm, which rules out any unimolecular multi-photon fluorescence mechanism (Figure S5). The photoluminescence excitation spectrum of the 418 nm emission possessed substantial contributions of PtOEP, corroborating the TTA-UC mechanism (Figure S6). The corresponding anti-Stokes shift was 5807 cm^−1^, typical of upconverted emission.

This UC emission involves two-photon processes, as seen from the quadratic dependence of its intensity on the photoexcitation power < 0.4 mW cm^−2^ (sky-blue region in Fig. 2b). The emission intensity became linearly proportional to the photoexcitation power > 0.4 mW cm^−2^ because the limiting step of TTA-UC changes from the bimolecular TTET or TTA processes to the unimolecular fluorescence transition (yellow region in Fig. 2b). The threshold photoexcitation power (i.e., 0.4 mW cm^−2^) is one order of magnitude smaller than those of the similar polymer UC nanoparticles of PtOEP and DPA^46,47^. The lower threshold photoexcitation power can be ascribed to the increased concentrations of the PtOEP and DPA dopants, specifically resulting in enhanced triplet–triplet energy transfer. The threshold photoexcitation power and the maximum upconversion efficiency remains invariant to repetitive photoexcitation (Figure S7), which rules out photodegradation. The Φ_UC_, which was determined using a rhodamine B standard^48^, was found to increase in proportion with the photoexcitation power, and reached a saturated value of 2.1% (Figure S9 and Methods). The Φ_UC_ remained 2.0% under the photocatalysis reaction condition described below (i.e., a distance of 10 cm from the photon source (Xe lamp) and a photoexcitation power of 2.8 mW cm^−2^). The threshold photoexcitation power and Φ_UC_ remained the same regardless of the presence of O_2_, indicating the high versatility of our system to the photoenzymatic reactions under aerobic conditions (Figure S8). The corresponding UC fluorescence brightness (*η*_UC_) was calculated to be 29 M^−1^ cm^−1^ with the relationship, *η*_UC_ = *ε*_ex_ × Φ_UC_, where *ε*_ex_ is the molar absorbance at 550 nm (1470 M^−1^ cm^−1^).

### Energy transfer from PS nanoparticles to *Cv*FAP

**Figure 3:**
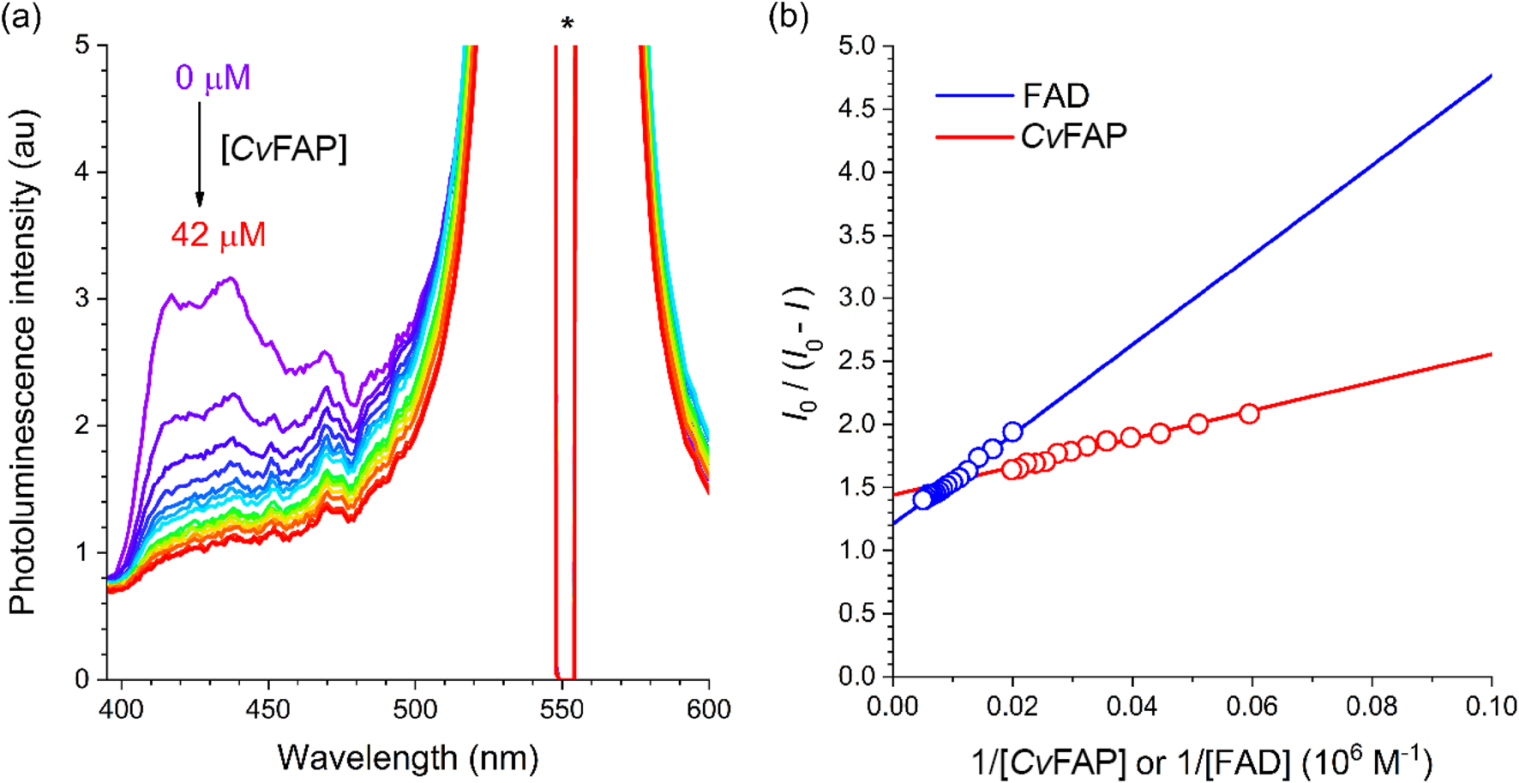
Photoactivation of a flavin-dependent photodecarboxylase. (**a**) Fluorescence titration results for the PtOEP:DPA@PS nanoparticle suspension (milli-Q water) recorded with increasing the concentration of a flavin-dependent photodecarboxylase (i.e., *Cv*FAP) (0–42 μM). The huge peak marked with an asterisk is the excitation beam (550 nm). See SI, Figure S11 for the titration results for free FAD. (**b**) Lehrer plot which depicts the corrected fluorescence intensity of the upconverted emission of the PtOEP:DPA@PS nanoparticle suspension (i.e., *I*_0_/(*I*_0_ − *I*), where *I*_0_ and *I* are the integrated values of the upconverted fluorescence intensities in the absence and presence, respectively, of *Cv*FAP or FAD) as functions of 1/[*Cv*FAP] and 1/[FAD], where [*Cv*FAP] and [FAD] are molar concentrations of *Cv*FAP and FAD, respectively.

To evaluate the photoactivation ability of PtOEP:DPA@PS, we performed fluorescence titration experiments. There was a substantial spectral overlap between the DPA emission and the FAD absorption spectra (Figure S5), which suggests an occurrence of resonance energy transfer from PtOEP:DPA@PS to the FAD-binding enzyme, *Cv*FAP. Indeed, the TTA-UC fluorescence intensity of PtOEP:DPA@PS decreases with the increased concentration of *Cv*FAP (0–42 μM) (Fig. 3a). Figure 3b depicts the corresponding *Cv*FAP fluorescence titration isotherm plotting *I*_0_/(*I*_0_ − *I*), where *I*_0_ and *I* are the integrated intensities of TTA-UC fluorescence in the absence and presence, respectively, of *Cv*FAP, as a function of an inverse of the added enzyme concentration (i.e., 1/[*Cv*FAP]). An apparent linear dependence between the two parameters was observed, which was analysed following the Lehrer’s modification of the Stern–Volmer equation (Lehrer equation, hereafter): *I*_0_/(*I*_0_ − *I*) = 1/*f* + 1/(*f* × *k*_Q_ × *τ* × [*Cv*FAP]). In this equation, *f* is the attenuation factor that accounts for the accessible fraction of the energy donor (i.e., DPA), *k*_Q_ is the bimolecular rate constant for quenching of TTA-UC fluorescence via energy transfer, and *τ* is the lifetime of the TTA-UC fluorescence determined through time-correlated single-photon-counting techniques (42 μs; Figure S10). Linear fitting of the titration isotherm to the Lehrer equations returned *f* and *k*_Q_ values of 0.69 and 3.1 × 10^9^ M^−1^ s^−1^, respectively.

TTA-UC fluorescence titration experiments were also conducted with free FAD, instead of *Cv*FAP (Figure S11). Free FAD produced the *f* value (0.83) greater than *f* (0.69) of *Cv*FAP, which indicated strong adhesion of *Cv*FAP at the surface of PtOEP:DPA@PS nanoparticles. This adhesion is beneficial for acceleration of energy transfer, as evidenced by the *k*_Q_ value (3.1 × 10^9^ M^−1^ s^−1^) of *Cv*FAP greater than that (0.81 × 10^9^ M^−1^ s^−1^) of FAD. The efficiency of energy transfer (Φ_ET_) from TTA-UC nanoparticles to *Cv*FAP was estimated with the relationship, Φ_ET_ = (*k*_Q_ × *τ* × [*Cv*FAP])/(1 + *k*_Q_ × *τ* × [*Cv*FAP]) to be 57%. This Φ_ET_ value is two-fold greater than that with free FAD (25%).

We also investigated energy transfer behaviors of free DPA (37 μM in THF) and DPA doped in PS nanoparticles (37 μM in PS) with FAD (Figure S2). Our analyses with the standard Stern–Volmer equation revealed that doped DPA exhibited an Φ_ET_ value (29%) greater than that (13%) of free DPA (Figure S12). This improvement was likely ascribed to delocalisation of DPA exciton within the PS nanoparticles, and demonstrated the benefit of the nanoparticle approach. Collectively, our spectroscopic investigations revealed a high photoactivation ability of the TTA-UC nanoparticles.

### Enzyme reactions under green light

The decarboxylation of ricinoleic acid (**1**) into (*Z*)-heptadec-9-en-7-ol (**2**) was used as a model reaction to examine application of TTA-UC for photoactivation of flavin-dependent photodecarboxylase (i.e., *Cv*FAP) (Fig. 1). After *Cv*FAP was added into the reaction medium containing 10 μM DPA in the form of ternary PtOEP:DPA@PS nanoparticles and 5 mM reaction substrates (**1**), green light (*λ* = 550 nm) was applied by a Xe lamp (Figure S13). The decarboxylation products (**2**) were detected to 0.29 mM in the reaction medium at t = 420 min by GC/MS analysis (Table 1 and Figure S14a). This result indicated that the green light was upconverted into blue light (*λ* = 418 nm) by the PtOEP:DPA@PS nanoparticles, which led to photoexcitation of the FAD of *Cv*FAP and subsequently decarboxylation of ricinoleic acid (**1**) into (*Z*)-heptadec-9-en-7-ol (**2**).

**Table 1:** Conversion of ricinoleic acid (**1**) into (*Z*)-heptadec-9-en-7-ol (**2**) by *Cv*FAP under green light.

Enzyme types	[Product (**2**)] (μM)		TON of DPA	TON of *Cv*FAP
	w/o PtOEP:DPA@PS	With PtOEP:DPA@PS		
Purified^a^	140 ± 28	290 ± 50	15	25
Whole-cells^b^	550 ± 45	980 ± 40	86	72
	550 ± 45	1300 ± 104	50	125

^a^The reactions were performed by the purified *Cv*FAP in the absence and presence of the PtOEP:DPA@PS nanoparticles, which are involved in light upconversion from green to blue light. Reaction conditions: *c*(Ricinoleic acid) = 5 mM, *c*(*Cv*FAP) = 6 μM, *c*(DPA) = 10 μM, illumination with green light (*λ* = 550 nm).

^b^The reactions were performed by the recombinant *E. coli* cells expressing *Cv*FAP in the absence and presence of the PS nanoparticles. Reaction conditions: *c*(Ricinoleic acid) = 5 mM, *c*(*E. coli*) = 7.2 g_CDW_ L^-1^ (*c*(*Cv*FAP) = ca. 6 μM), *c*(DPA) = 5 μM (up) or 15 μM (down). The TONs were calculated based on the product concentration at t = 420 min.

The fatty alcohol (i.e., (*Z*)-heptadec-9-en-7-ol (**2**)) was also observed to 0.14 mM in the reaction medium without the nanoparticles (Figure S14b), suggesting that FAD of *Cv*FAP might be activated by green light irradiated by the Xe lamp. The turnover number (TON) of DPA in the nanoparticles and the enzyme was calculated to 15 and 25, respectively (Table 1), meaning that the light upconversion was 15 times achieved per molecule of DPA.

The TON of *Cv*FAP (Table 1) was low in the light upconversion system as compared to the blue light-based reaction system^18^. One of the reasons may include formation of the reactive oxygen species (ROS) during light upconversion^41^, which may cause deactivation of the enzymes via oxidation of the sulfur-containing amino acids (e.g., cysteine 432, which is involved in catalysis)^49^.

### Whole-cell reactions under green light

Aiming at improving the enzyme reaction rates and TONs under green light, recombinant *E. coli* cells, which provide the ROS quenching systems (e.g., glutathione peroxidases and catalases) to intracellular enzymes, were used as biocatalysts. After the recombinant *E. coli* cells expressing *Cv*FAP were added into the reaction medium containing 5 or 15 μM DPA in the form of PtOEP:DPA@PS nanoparticles and 5 mM reaction substrate (**1**), green light (*λ* = 550 nm) was irradiated by the Xe lamp (Figure S13).

**Figure 4:**
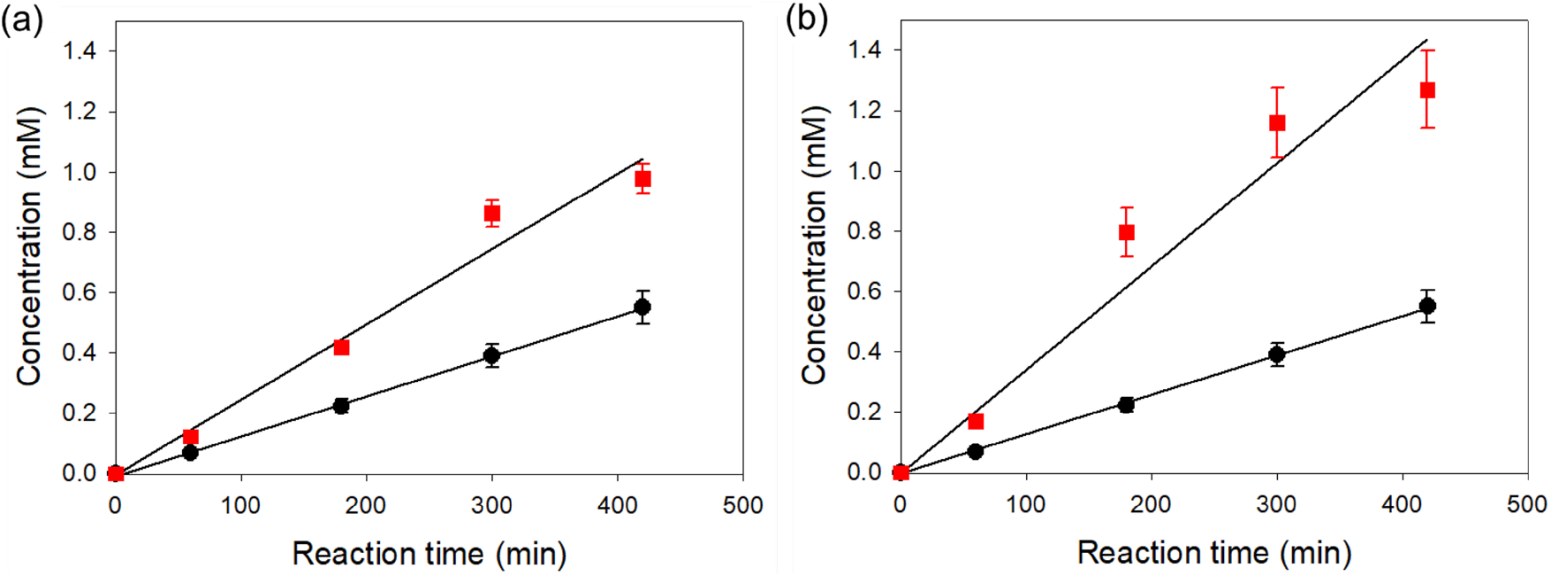
Time course of photodecarboxylations. Decarboxylation of ricinoleic acid (**1**) into (*Z*)-heptadec-9-en-7-ol (**2**) was carried out by the recombinant *E. coli* cells expressing *Cv*FAP under green light (*λ* = 550 nm). The reactions were performed in the absence (filled black circle) and presence (filled red square) of the PtOEP:DPA@PS nanoparticles, which are involved in light upconversion from green to blue light. Reaction conditions: *c*(Ricinoleic acid) = 5 mM, *c*(Cat) = 7.2 g_CDW_ L^-1^, *c*(DPA) = 5 μM (**a**) or 15 μM (**b**).

When 5 μM DPA was used for the light upconversion, (*Z*)-heptadec-9-en-7-ol (**2**) was produced to a rate of 2.5 μM/min, while the fatty alcohol (**2**) was produced to 1.4 μM/min without PS nanoparticles (Fig. 4a). This result indicated that (*Z*)-heptadec-9-en-7-ol (**2**) was produced to 1.1 μM/min via the light upconversion. Since the product concentrations reached 0.98 and 0.55 mM at t = 420 min in the presence and absence of the nanoparticles, the TONs of DPA and *Cv*FAP at t = 420 min were estimated to 86 and 72, respectively, which are significantly greater than those of isolated enzyme reaction systems (Table 1). These results suggested that the FADs in the core of *Cv*FAP enzymes, which were located in cytoplasm of the recombinant *E. coli* cells, had been quite efficiently excited by the PtOEP-DPA upconversion systems inside the poly(styrene) nanoparticles (Figure S13).

The increase of DPA concentration in PtOEP:DPA@PS nanoparticles to 15 μM led to formation of (*Z*)-heptadec-9-en-7-ol (**2**) to a rate of 3.2 μM/min (Fig. 4b). On the other hand, the fatty alcohol (**2**) was produced to 1.4 μM/min in the buffer without the PS nanoparticles, as in the experiment shown in Fig. 4b. In addition, the biotransformations in the buffer containing polystyrene nanoparticles only or DPA@PS nanoparticles only showed slightly lower product formation as compared to the biotransformation in the buffer without anything (Figure S15). Thereby, it was assumed that the target product was produced in the reaction medium to at least 1.8 μM/min via the light upconversion. The reaction rate was approximately 60% greater than that of the reaction system including 5 μM DPA as annihilator. Increased DPA concentrations in PtOEP:DPA@PS nanoparticles did not alter the TTA-UC behaviors (Figure S16). Thereby, it was assumed that the DPA concentrations have an influence on the photobiocatalytic reaction rates but did not linearly correlate.

The biotransformation of ricinoleic acid (**1**) into (*Z*)-heptadec-9-en-7-ol (**2**) was also carried out by the recombinant *E. coli* cells expressing *Cv*FAP under blue light (*λ* = 450 nm). The reaction rate was significantly greater than under green light (*λ* = 550 nm) (Figure S17), indicating that the PtOEP:DPA@PS nanoparticles need to be further improved. Thereby, the future study will focus on improvements of the TTA-UC system for enzymatic reactions in aqueous reaction systems.

Not only light-induced natural enzyme reactions but also light-dependent promiscuous enzymatic conversions have been extensively investigated for the environmentally benign synthesis of chemicals^1–5,8–10,15,18–20^ and fuels^21–23^. However, as flavin catalysts utilize only a part of the wavelength spectrum of visible light (e.g., *λ* = 450 nm), a significant part of the energy provided by visible light remains unused. Another issue of photobiocatalysis comprises the rather poor light penetration in traditional glass-batch reactors and external illumination^50,51^. Particularly blue light (*λ* = 450 nm) typically penetrates no more than a few millimeters to centimeters (especially in optically dense reaction mixtures). The problem should become more serious in scale-up of the photobiocatalysis. This study demonstrated that the PtOEP:DPA@PS-based TTU-AC system can be used to partially solve the poor penetration of blue light in photobiocatalytic reactors, by enabling the enzymes to use green light (*λ* = 550 nm) as a light source, which is capable of penetrating deeper into the core of bioreactors without damaging the enzyme biocatalysts.

## Conclusions

This study demonstrated the effectiveness of the upconversion strategy toward steering photobiocatalysis. Upconverted fluorescence emission from TTA-UC poly(styrene) nanoparticles doped with the PtOEP photosensitiser and the DPA annihilator could photoactivated FAD and FAD-bound enzymes in aqueous solutions. Combination of TTA-UC nanoparticles and bacterial cells expressing *Cv*FAP in aqueous reaction systems allowed to catalyse decarboxylation of fatty acids into secondary fatty alcohols under green light. The results will provide useful guidance to synthetic application of photobiocatalysis.

## Supplementary Information

Supplementary Information.

## Data Availability

The datasets used and/or analysed during the current study available from the corresponding author on reasonable request.
