# Supplementary material for: Triplet–triplet annihilation-based photon-upconversion to broaden the wavelength spectrum for photobiocatalysis
Source: Sci Rep. 2022 Jun 7;12:9397. doi: 10.1038/s41598-022-13406-8 (PMC9174481; doi:10.1038/s41598-022-13406-8)
Supplement: Supplementary file 1 — Supplementary Information. [file 41598_2022_13406_MOESM1_ESM.docx]

**Triplet-Triplet Annihilation-based Photon-Upconversion to Broaden the Wavelength Spectrum for Photobiocatalysis**

Se-Yeun Hwang,^1, ‡^ Dayoon Song,^2, ‡^ Eun-Ji Seo,^1^ Frank Hollmann,^3^ Youngmin You,^2, *^ and Jin-Byung Park^1,*^

^1^ Department of Food Science & Biotechnology, Ewha Womans University, Seoul 03760, Republic of Korea.

^2^ Division of Chemical Engineering and Materials Science, and Graduate Program in System Health Science and Engineering, Ewha Womans University, Seoul 03760, Republic of Korea.

^3^ Department of Biotechnology, Delft University of Technology, Van der Maasweg 9, 2629HZ Delft, The Netherlands.

^‡^These authors are equally contributed first authors.

^*^E-mail: odds2@ewha.ac.kr; jbpark06@ewha.ac.kr

**Materials and Methods**

**Chemicals and Reagents.** 9,10-Diphenylanthracene (DPA), ricinoleic acid, flavin adenine dinucleotide (FAD) disodium salt hydrate, and *N*-methyl-*N*-(trimethylsilyl)trifluoroacetamide (TMS) were obtained from Tokyo Chemical Industry Co. (Tokyo, Japan). Platinum(II) octaethylporphyrin (PtOEP), poly(styrene), isopropyl-β-D-thiogalactopyranoside (IPTG), pyridine, and kanamycin were purchased from Sigma (St. Louis, MO, USA). Dimethyl sulfoxide (DMSO), ethyl acetate, methanol, and glycerol were provided by Duksan Pure Chemical Co. (Ansan, Korea). Terrific broth was purchased from BD Difco^TM^ (NJ, USA). Tetrahydrofuran (THF) was purchased from Junsei Chemical Co. (Tokyo, Japan).

**Field-Emission Scanning Electron Microscopy.** An aliquot of an aqueous solution (milli-Q water) containing PtOEP:DPA@PS nanoparticles was placed onto SiO2 wafer, which was dried in vacuo overnight. Platinum was subsequently deposited onto the sample using a Hitachi, MC1000 ion sputter coater at 15 mA for 60 s. The surface of the particles was visualized employing a Hitachi, SU8220 field-emission scanning electron microscope.

**Dynamic Light Scattering Experiments**. The distribution of the diameter of PtOEP:DPA@PS nanoparticles were determined through dynamic light scattering experiments by using a Photal Otsuka Electronics, ELS-Z1000 instrument at room temperature. Data analyses were performed with employing the software provided by the manufacturer.

**Determination of Doping Concentrations**. The doping concentrations of PtOEP and DPA in PS nanoparticles were determined by UV−vis absorption spectroscopy. The UV−vis absorption spectrum of the PtOEP:DPA@PS nanoparticle suspension (milli-Q water) contained significant scattering, as well as the characteristic absorption bands of PtOEP and DPA. To quantify the scattering signals, we independently prepared PS nanoparticles without the dopants. The UV−vis absorption spectrum of PS nanoparticles was recorded. The difference between the UV−vis absorption spectra of PtOEP:DPA@PS and PS nanoparticles was taken as the true absorption spectrum of the PtOEP:DPA@PS nanoparticles. The doping concentrations of PtOEP and DPA were determined with the Beer−Lambert’s law, using the molar absorbance (*ε*) values of PtOEP (*ε*(539 nm)_PtOEP_ = 26640 M^−1^ cm^−1^ and *ε*(408 nm)_PtOEP_ = 9840 M^−1^ cm^−1^) and DPA (*ε*(408 nm)_DPA_ = 1270 M^−1^ cm^−1^). Note that both PtOEP and DPA absorb the 408 nm light. Therefore, absorbance at 539 (*Abs*(539 nm)) and 408 nm (*Abs*(408 nm)) has the following relationships:

*Abs*(539 nm) = *ε*(539 nm)_PtOEP_ × *b* × *c*_PtOEP_ (eq 1)

*Abs*(408 nm) = [*ε*(408 nm)_PtOEP_ × *c*_PtOEP_ + *ε*(408 nm)_DPA_ × *c*_DPA_] × *b* (eq 2)

In eqs 1 and 2, *b* is the beam path length (1 cm), *c*_PtOEP_ is the molar concentration of PtOEP, and *c*_DPA_ is the molar concentration of DPA. The absorbance of the PtOEP:DPA@PS nanoparticles at wavelengths 539 and 408 nm were 0.094 and 0.20, which returned *c*_PtOEP_ and *c*_DPA_ values to be 3.5 μM and 130 μM, respectively. Finally, the encapsulation efficiencies for PtOEP and DPA were calculated to be 29% and 24%, respectively, using the feed concentrations (PtOEP, 12 μM; DPA, 540 μM).

**Steady-state Photoluminescence Spectroscopy**. The PtOEP:DPA@PS nanoparticle suspension in milli-Q water was used for fluorescence experiments, unless otherwise specified. Solutions of DPA, PtOEP and DPA were prepared in THF at concentrations of 10 μM prior to the measurements. The samples were transferred to a quartz cuvette (Hellma, beam path length = 1.0 cm). Photoluminescence spectra were obtained using a Photon Technology International, Quanta Master 400 scanning spectrofluorometer at 298 K. Samples were photoexcited at wavelengths of 550 and 394 nm. The photoluminescence excitation spectrum was obtained at an emission peak wavelength of 418 nm. The photoexcitation power was varied in the range 0.2−7.0 mW cm^−2^ by changing the excitation slits, and was quantified with a Newport, 843-R optical power meter. The excitation power density was calculated by dividing the photoexcitation power with a beam area (0.79 cm^2^). The emission was collected in the range 380−750 nm under an air-equilibrated condition.

**Steady-state UV−vis Absorption Spectroscopy**. UV–vis absorption spectra of solutions were collected using an Agilent, Cary 300 spectrophotometer at 298 K. The solutions were prepared at a concentration of 10 μM prior to the measurements, unless otherwise stated. The solution was delivered into a quartz cell (Hellma, beam path length = 1.0 cm).

**Determination of Upconversion Fluorescence Quantum Yield**. The upconversion fluorescence quantum yield (Φ_UC_) was determined for the PtOEP:DPA@PS nanoparticle suspension (milli-Q water), using the following relationship:

$$\Phi_{UC}=\Phi_{std}\times\frac{{Abs}_{std}}{{Abs}_{UC}}\times\frac{I_{UC}}{I_{std}}\times\left( \frac{n_{UC}}{n_{std}} \right)^{2} (\mathrm{eq} 3)$$

In eq 3, Φ_std_ is the fluorescence quantum yield of the rhodamine B standard (0.5; ethanol), *Abs*_std_ and *Abs*_UC_ are absorbance values at 550 nm of the standard and nanoparticles, *I*_UC_ and *I*_std_ are the integrated values of the upconverted fluorescence spectra of the nanoparticles and the standard, and *n*_UC_ and *n*_std_ are refractive indices of water (1.33) and ethanol (1.36). The Φ_UC_ values were determined with varying the photoexcitation power in the range of 0.2−7.0 mW cm^−2^.

**Fluorescence Titration Experiments**. Photoluminescence spectra of PtOEP:DPA@PS nanoparticle suspension (milli-Q water) were recorded with the addition of *Cv*FAP (0−42 μM) and FAD (0−200 μM). A stock solution of 850 μM *Cv*FAP in an aqueous solution buffered to pH 8.0 (25 mM Tris-HCl) was used. In the case of FAD, a 2.0 mM FAD solution was freshly prepared in milli-Q water. The fluorescence spectrum of each solution was recorded and the fluorescence intensity (*I*) was calculated by integrating the spectrum from 395 nm to 475 nm. A titration isotherm plotting the corrected fluorescence intensity (*I*_0_ / (*I*_0_ − *I*), where *I*_0_ and *I* are the integrated fluorescence intensities in the absence and presence, respectively, of *Cv*FAP or FAD) as a function of an inverse of the added concentration of the quencher (i.e., 1/[Q]; Q refers to *Cv*FAP and FAD) was constructed and fitted to the following Lehrer’s equation to return the bimolecular quenching rate (*k*_Q_):

$$\frac{I_{0}}{I_{0}-I}=\frac{1}{f}+\frac{1}{f\times k_{Q}\times\tau\times[Q]} (\mathrm{eq} 4)$$

In eq 4, *f* is the attenuation factor which accounts for the fraction of DPA accessible to the quencher and *τ* is the fluorescence lifetime of the DPA emission of the PtOEP:DPA@PS nanoparticle suspension (42 μs). Linear fitting of the titration isotherm to eq 4 yielded *k*_Q_ values for *Cv*FAP (3.1 × 10^9^ M^−1^ s^−1^) and FAD (8.1 × 10^8^ M^−1^ s^−1^). This method could not be applied for the FAD titration isotherms for the DPA@PS nanoparticle suspension (milli-Q water) and the DPA solution (DMSO), because they produced *f* > 1. We, thus, employed the the Stern−Volmer equation to determine the *k*_Q_ values. Finally, the *k*_Q_ values introduced the following relationship to give the energy transfer efficiency (Φ_ET_):

$$\Phi_{\mathrm{ET}}=\frac{k_{Q}\times\tau\times[Q]}{1+k_{Q}\times\tau\times[Q]} (\mathrm{eq} 5)$$

Note that *Φ*_ET_ depends on [Q], so we reported *Φ*_ET_ values at a 10 μM concentration of the quencher.

**Transient Photoluminescence Measurements**. Photoluminescence decay traces were acquired based on time-correlated single-photon-counting (TCSPC) techniques by using a PicoQuant, FluoTime 200 instrument. A 377 nm diode laser (PicoQuant; pulse energy = 35 pJ) with a repetition rate of 125 kHz was used as the excitation source. The transient signals at 418 nm were obtained by using an automated motorized monochromator. The decay profiles were analyzed (OriginPro 8.0, OriginLab) by using a biexponential decay model.

**Preparation of the Photodecarboxylase from *Chlorella variabilis* NC64A.** The photodecarboxylase of *C. variabilis* NC64A (*Cv*FAP) was prepared based on our previous studies.^1, 2^ Briefly, the recombinant *Escherichia coli* BL21 (DE3) cells harboring the pET28a-His-TrxA-*Cv*FAP plasmid were pre-cultured in terrific broth (TB) medium with kanamycin at shaking incubator (37 ℃, 250 rpm).^1, 2^ The pre-cultures were then inoculated into main cultures (50 mL TB medium + kanamycin in 500 mL flasks) and were incubated at 37 ℃ shaking incubator (250 rpm). When OD_600_ reached between 0.6-0.8, the *Cv*FAP gene expression was induced by 0.5 mM IPTG and the cells were additionally incubated at 20 °C, 200 rpm for 20 h.

The *Cv*FAP was purified on a basis of our earlier studies.^1, 3^ Shortly, the recombinant cells were harvested by centrifugation and washed with 50 mM Tris-HCl buffer (pH 8). After lysis of the cells by sonication, the cell lysate was subjected to Ni-NTA column affinity chromatography. The *Cv*FAP concentration was estimated by the measurement of UV absorbance at 280 nm.

**Photodecarboxylation by *Cv*FAP.** Decarboxylation of fatty acids by *Cv*FAP was performed on a basis of our previous studies.^1, 4^ For enzyme reactions, 6 μM of the purified *Cv*FAP and 5 mM of ricinoleic acid were added to 50 mM Tris-HCl buffer (pH 8.0 with 30% (v/v) DMSO as a cosolvent). After addition of 10 μM DPA in the form of PtOEP:DPA@PS nanoparticle to the reaction medium, green light (*λ* = 550 nm) with light intensity of 1.1 mW cm^−2^ was illuminated by Xenon lamp MAX 303 and stirred at 37 ℃ for 420 min. Reaction condition: *c*(Ricinoleic acid) = 5 mM, *c*(*Cv*FAP) = 6 μM, *c*(DPA) = 10 μM, 50 mM Tris-HCl buffer (pH 8.0, with 30% (v/v) DMSO), illumination with green light (*λ* = 550 nm), total reaction volume = 2 mL.

For the whole cell reactions, the recombinant *E. coli* BL21 (DE3) pET28a-His-TrxA-*Cv*FAP were harvested by centrifugation and resuspended in 50 mM Tris-HCl buffer (pH 8.0). After addition of 5 mM of ricinoleic acid and 5 to 15 μM DPA in the form of PtOEP:DPA@PS nanoparticle to the reaction medium, green light (*λ* = 550 nm) was illuminated by Xenon lamp MAX 303 and stirred at 37 ℃ for 420 min. Reaction conditions: *c*(Ricinoleic acid) = 5 mM, *c*(*E. coli*) = 7.2 g_CDW_ L^-1^ (*c*(*Cv*FAP) = ca. 6 μM), *c*(DPA) = 5 μM or 15 μM, 50 mM Tris-HCl buffer (pH 8.0), illumination with green light (*λ* = 550 nm), total volume = 2 mL.

**Gas chromatography/mass spectrometry (GC/MS) analysis of the products.** Concentrations of the reactants and products (e.g., ricinoleic acid (**1**) and (*Z*)-heptadec-9-en-7-ol (**2**)) were estimated based on our previous studies.^4, 5^ Briefly, after the reactants and products were separated by twice volume of ethyl acetate containing 2.5 g/L or 5 g/L of palmitic acid as an internal standard, the organic extracts were derivatived with TMS. The samples were analyzed by GC/MS (HP-5MS, column 30 m $\times$ 0.25 mm $\times$ 0.25 µm).

The turnover numbers (TONs) of DPA and *Cv*FAP during catalysis were calculated, which are the division of concentrations of the products at t = 420 min by the concentrations of DPA or *Cv*FAP.^1^

TON of DPA $=\frac{{[product]}_{420 \min}}{[DPA]}$ (eq 6)

TON of *Cv*FAP $=\frac{{[product]}_{420 \min}}{[CvFAP]}$ (eq 7)

**Supplementary Figures**


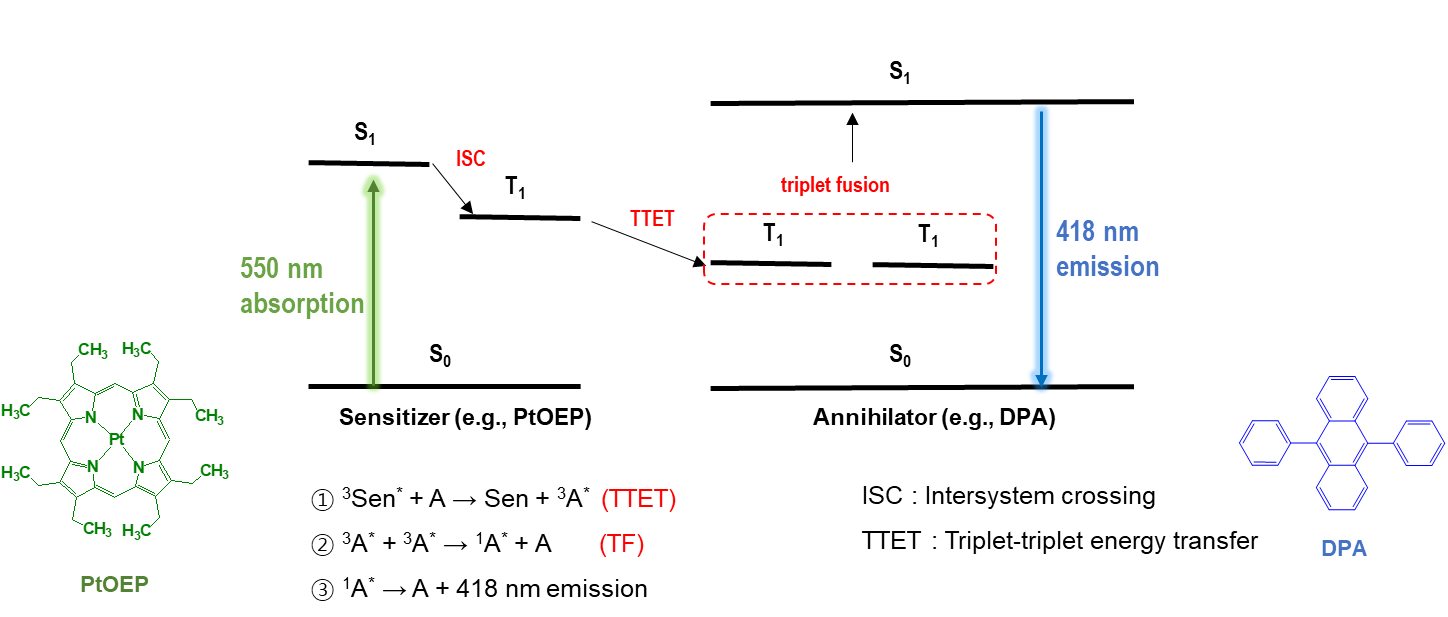


**Figure S1**. Overall concept of the triplet-triplet annihilation-based upconversion (TTA-UC). TTA-UC is a photochemical reaction that generates one higher energy photon from two or more low energy photons. TTA-UC is based on energy transfer and fusion between photosensitizer and annihilator chromophores. The reaction begins with the absorption of low energy photon by photosensitizer (e.g., PtOEP), and generates long-lived triplet excited state through intersystem crossing. The triplet-triplet energy transfer (TTET) between this triplet excited state to a ground state of annihilator (e.g., DPA) leads to the generation of long-lived triplet excited annihilator. Two triplet excited state of annihilators then perform triplet fusion that leads to a high-energy singlet state. As the singlet excited state of annihilator returns to the ground state, it emits one single photon as a fluorescence.^6^


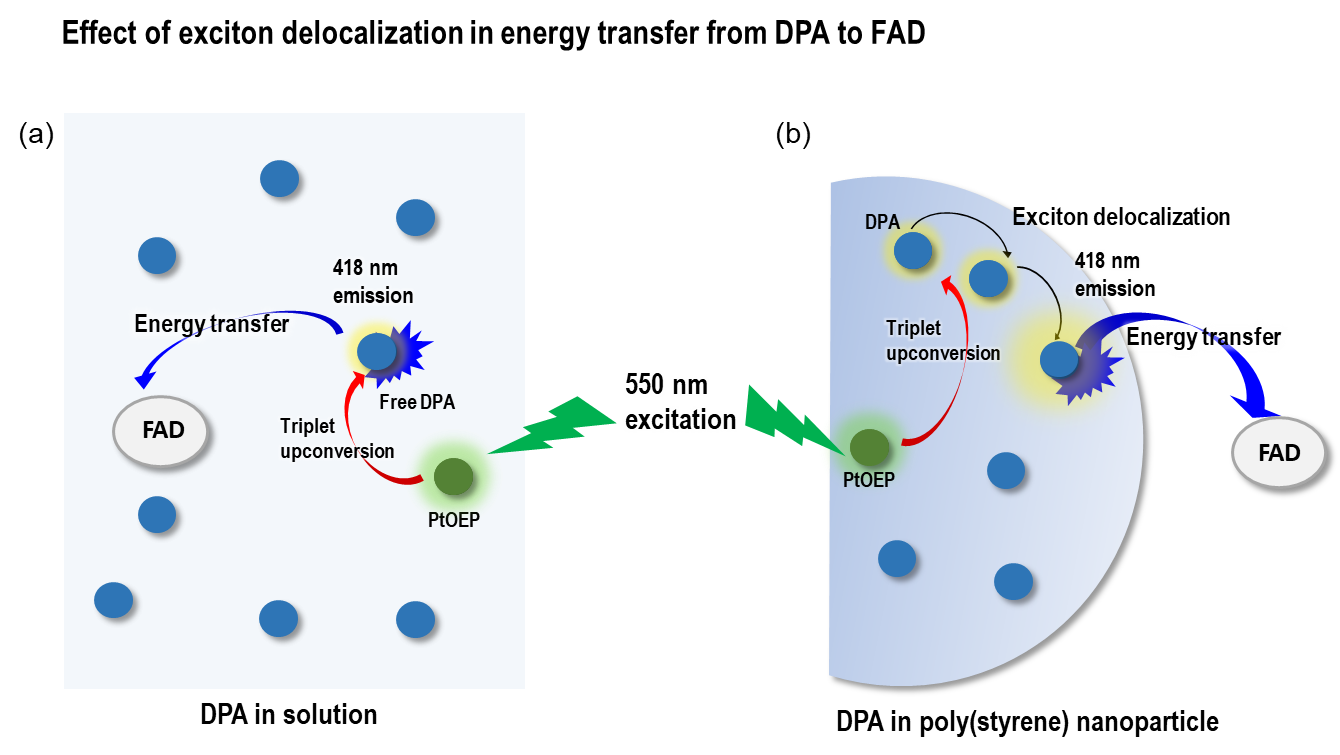


**Figure S2**. Light upconversion and energy transfer via free DPA in solution (a) and encapsulated DPA in the poly(styrene) (PS) nanoparticles (b). Exciton delocalization in energy transfer from DPA to FAD may take place in the PS nanoparticle system.


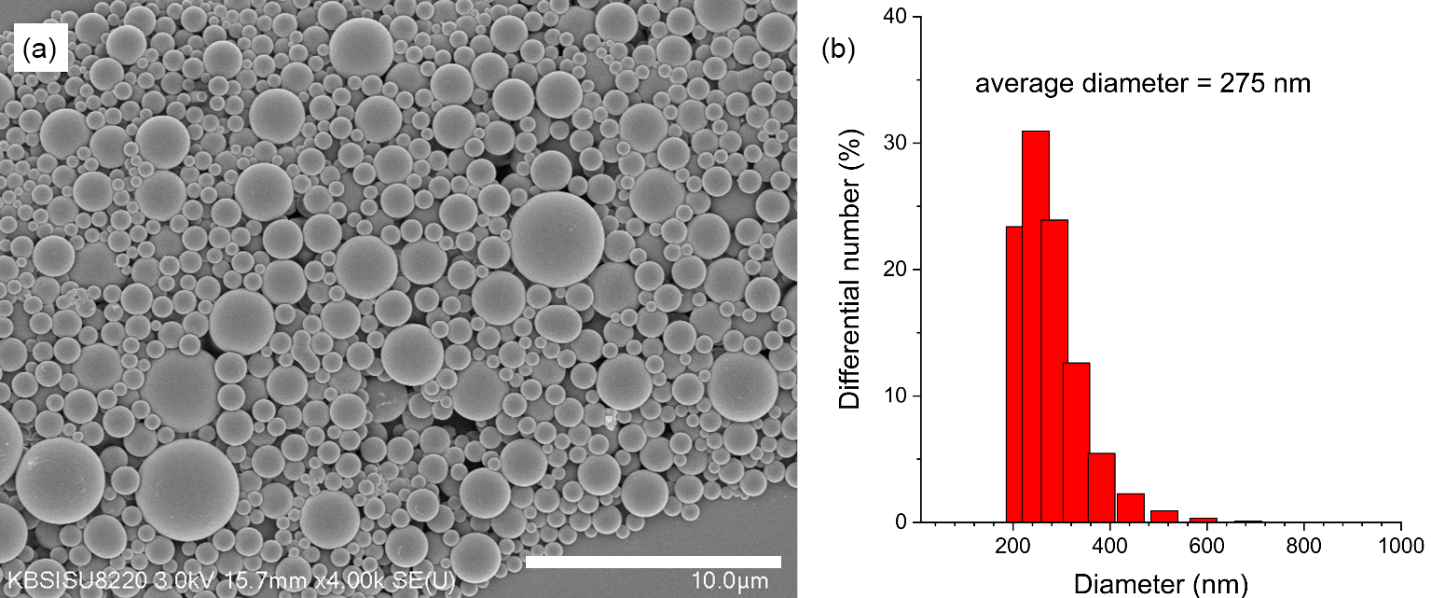


**Figure S3**. (a) Field-emission scanning electron micrograph (3 kV) of PtOEP:DPA@PS nanoparticles. The scale bar is 10 μm. (b) Distribution of the diameter of PtOEP:DPA@PS nanoparticles determined by dynamic light scattering experiments.


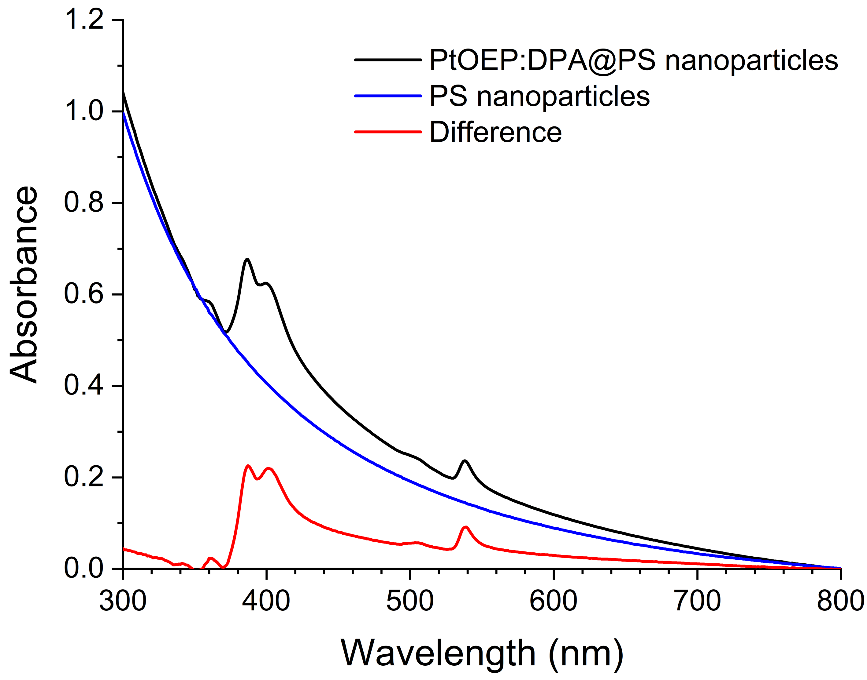


**Figure S4**. UV−vis absorption spectra for the PtOEP:DPA@PS nanoparticles (black) and PS nanoparticles without PtOEP and DPA dopants (blue). The red curve is the difference spectrum of the black and blue curves.


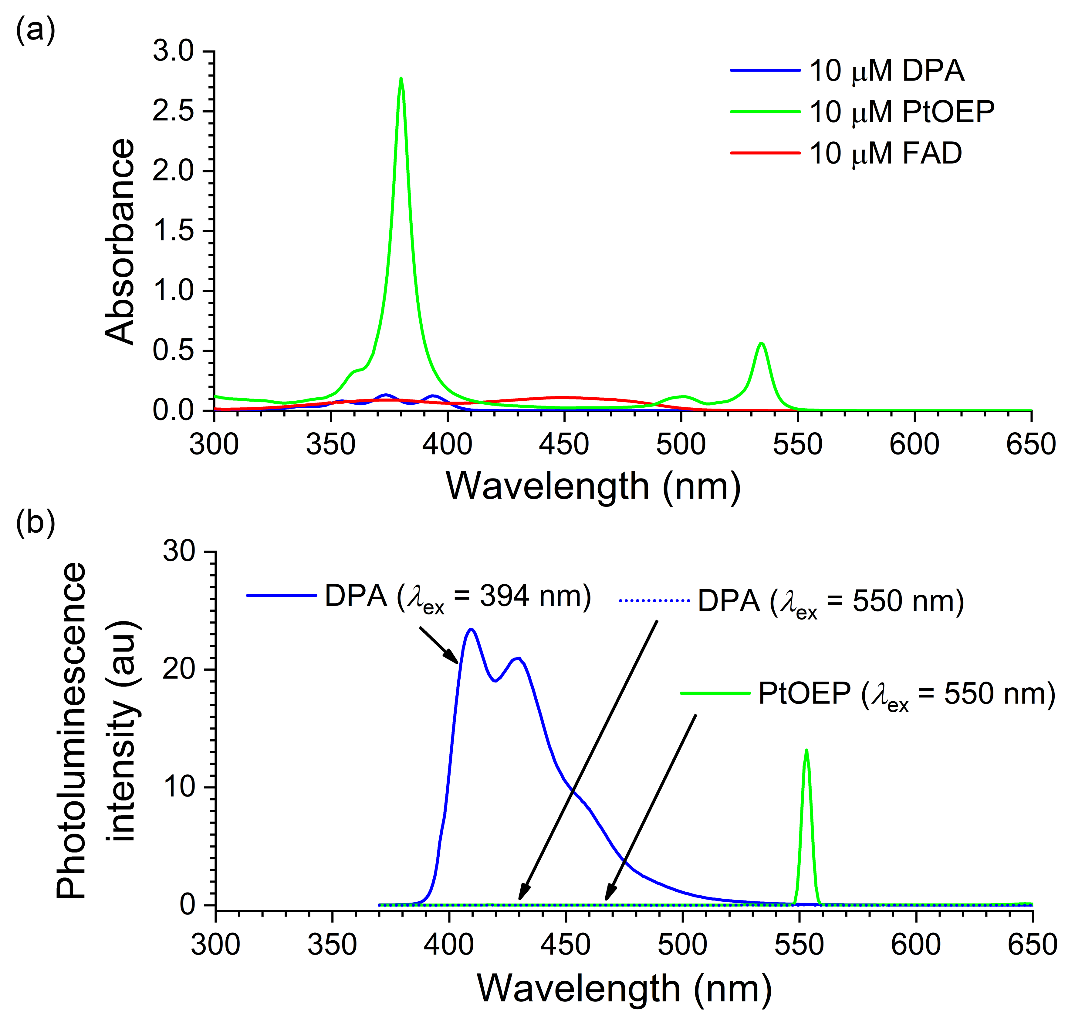


**Figure S5**. (a) UV−vis absorption spectra of 10 μM DPA (THF), 10 μM PtOEP (THF) and 10 μM FAD (milli-Q water). (b) Photoluminescence spectra of 10 μM DPA (THF) and 10 μM PtOEP (THF) recorded at varied photoexcitation wavelengths.


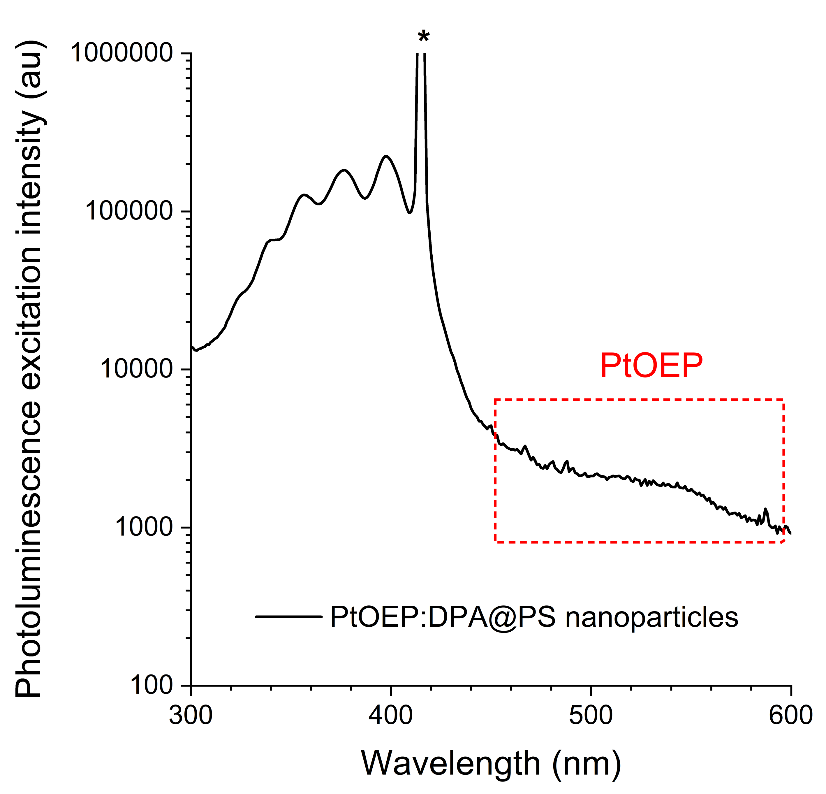


**Figure S6**. Photoluminescence excitation spectrum of the PtOEP:DPA@PS nanoparticle suspension (milli-Q water) recorded at the emission peak wavelength of 418 nm. The red box indicates the excitation of PtOEP. The peak marked with an asterisk is the emission beam (i.e., 418 nm).





**Figure S7**. Double-logarithmic plots of the photoluminescence intensity of TTA-UC nanoparticle (i.e., PtOEP:DPA@PS nanoparticle) suspension as functions of the photoexcitation power. The photoluminescence intensity was recorded in triplicate for an identical suspension upon the photoexcitation of PtOEP at a wavelength of 550 nm. The threshold photoexcitation power (*I*_th_) is included.


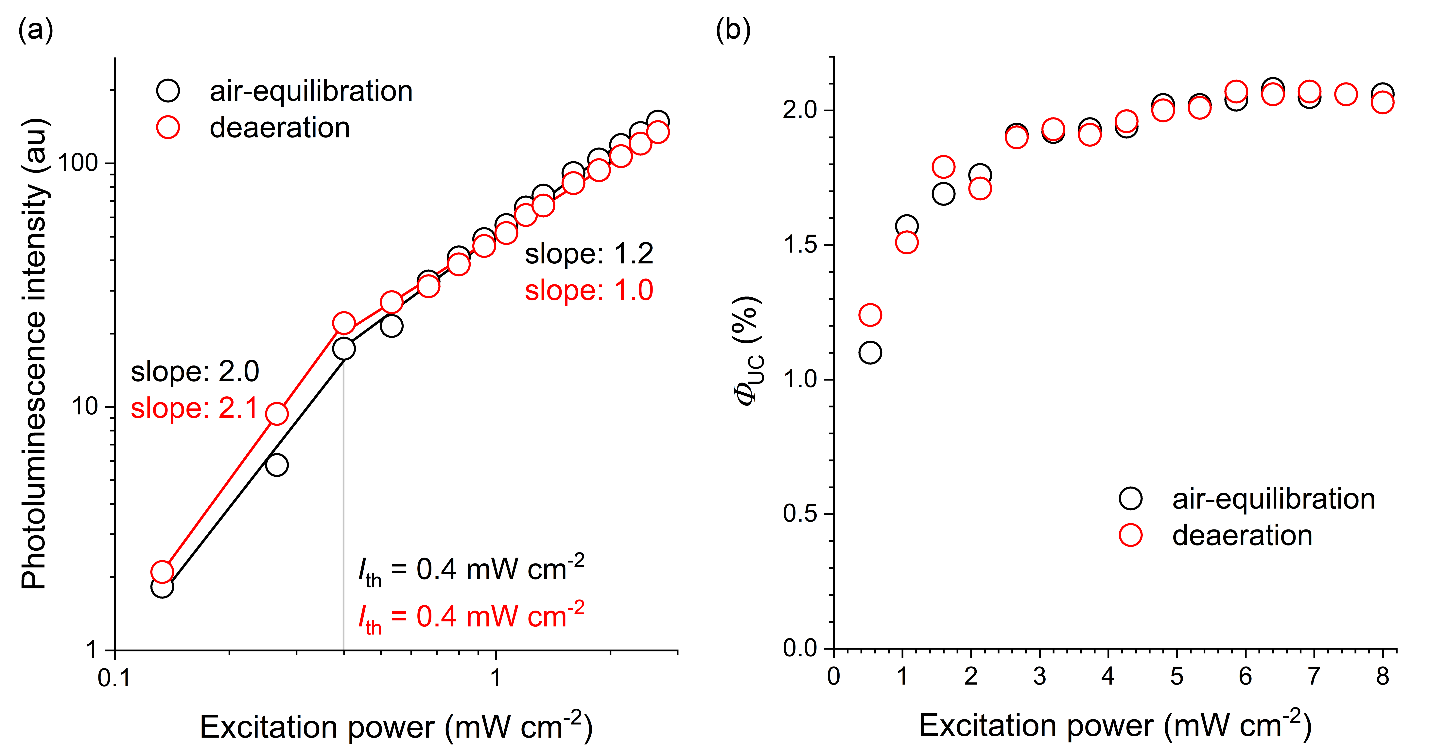


**Figure S8**. (a) Double-logarithmic plots of the photoluminescence intensity of TTA-UC nanoparticle (i.e., PtOEP:DPA@PS nanoparticle) suspension in the absence and presence of O_2_ as functions of the photoexcitation power. The threshold photoexcitation power (*I*_th_) is included. (b) The corresponding upconversion fluorescence quantum yields.


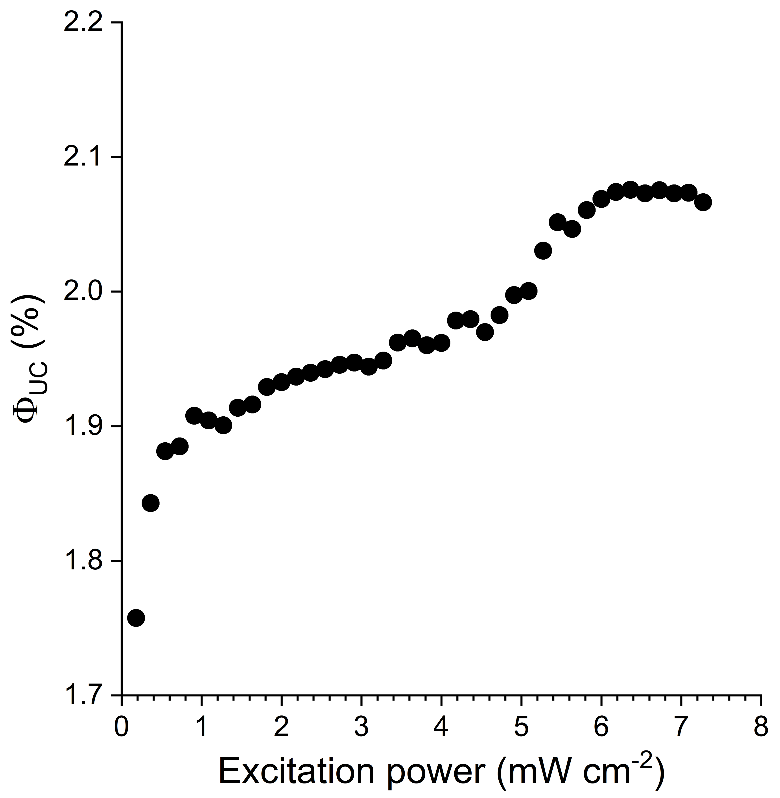


**Figure S9**. Upconversion fluorescence quantum yields the PtOEP:DPA@PS nanoparticle suspension (milli-Q water) obtained with varied photoexcitation power at a wavelength of 550 nm.


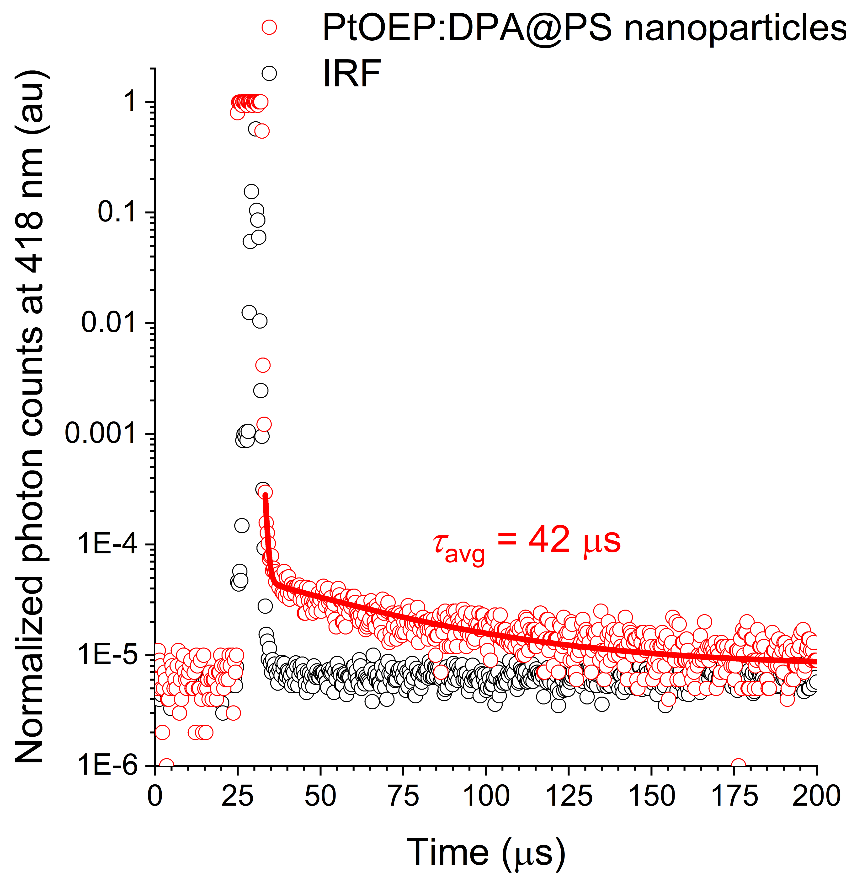


**Figure S10**. Photoluminescence decay traces (red symbols) of the PtOEP:DPA@PS nanoparticle suspension (milli-Q water) recorded at *λ*_em_ = 418 nm after pulsed laser excitation at 377 nm (temporal resolution = 320 ns). The solid curve is a non-linear least-squares fit of the data to a biexponential decay model. The black symbols are the instrumental response function.


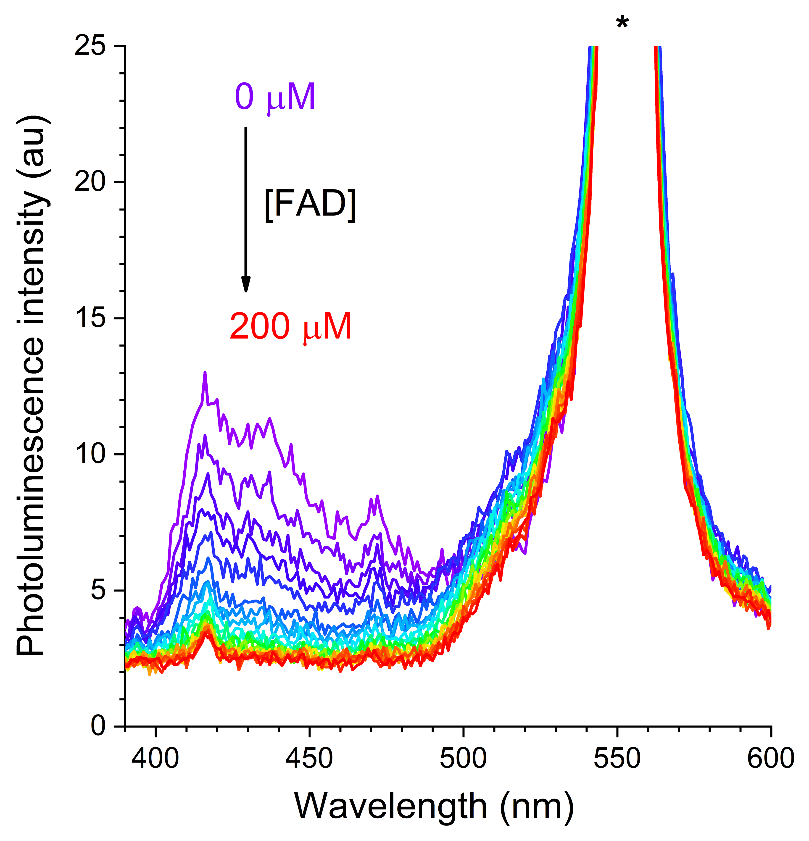


**Figure S11**. Fluorescence titration results for the PtOEP:DPA@PS nanoparticle suspension (milli-Q water) recorded with increasing the concentration of FAD (0−200 μM). The huge peak marked with an asterisk is the excitation beam (550 nm).


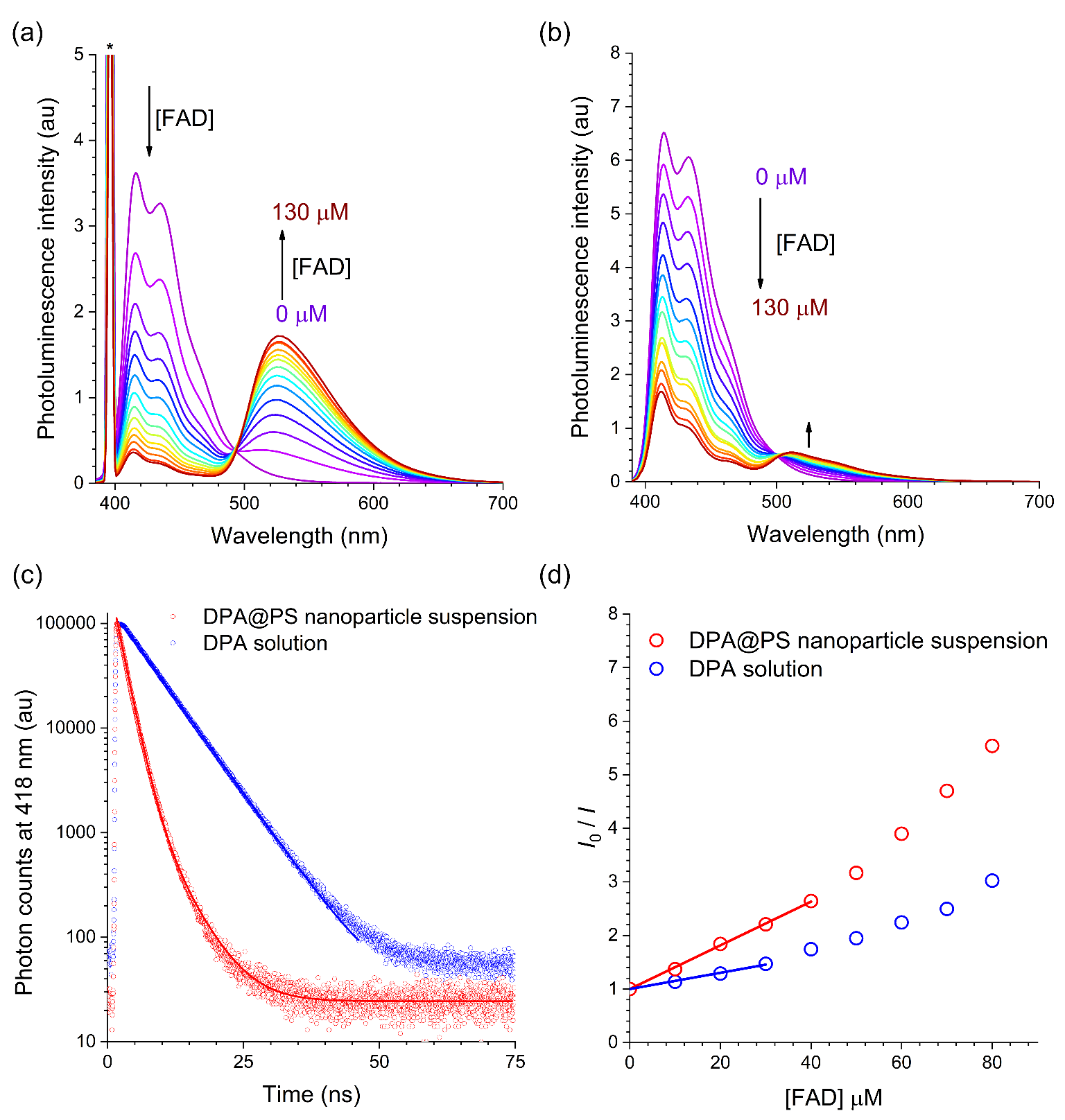


**Figure S12**. (a) Fluorescence titration results for the DPA@PS nanoparticle suspension (milli-Q water) recorded with increasing the concentration of FAD (0−130 μM). The peak marked with an asterisk is the excitation beam (394 nm). (b) Fluorescence titration results for 37 μM DPA (DMSO) recorded with increasing the concentration of FAD (0−130 μM). (c) Photoluminescence decay traces of the DPA emissions (*λ*_em_ = 418 nm) of the DPA@PS nanoparticle suspension (red symbols, milli-Q water) and 37 μM DPA (blue symbols, DMSO) recorded after pulsed laser excitation at 377 nm. The solid lines are non-linear least-squares fits to a biexponential decay model. (d) Fluorescence FAD titration isotherms of DPA@PS nanoparticle suspension (milli-Q water) and 37 μM DPA (DMSO) plotting the corrected fluorescence intensity (*I*_0_/*I*) as functions of the added concentration of FAD ([FAD]). The titration isotherms were fitted to the Stern−Volmer equation that accounts only for the dynamic quenching, *I*_0_/*I* = 1 + *k*_Q_*τ* [FAD], where *τ* values are 1.7 ns and 6.0 ns for the DPA@PS nanoparticle suspension and the DPA solution, respectively.


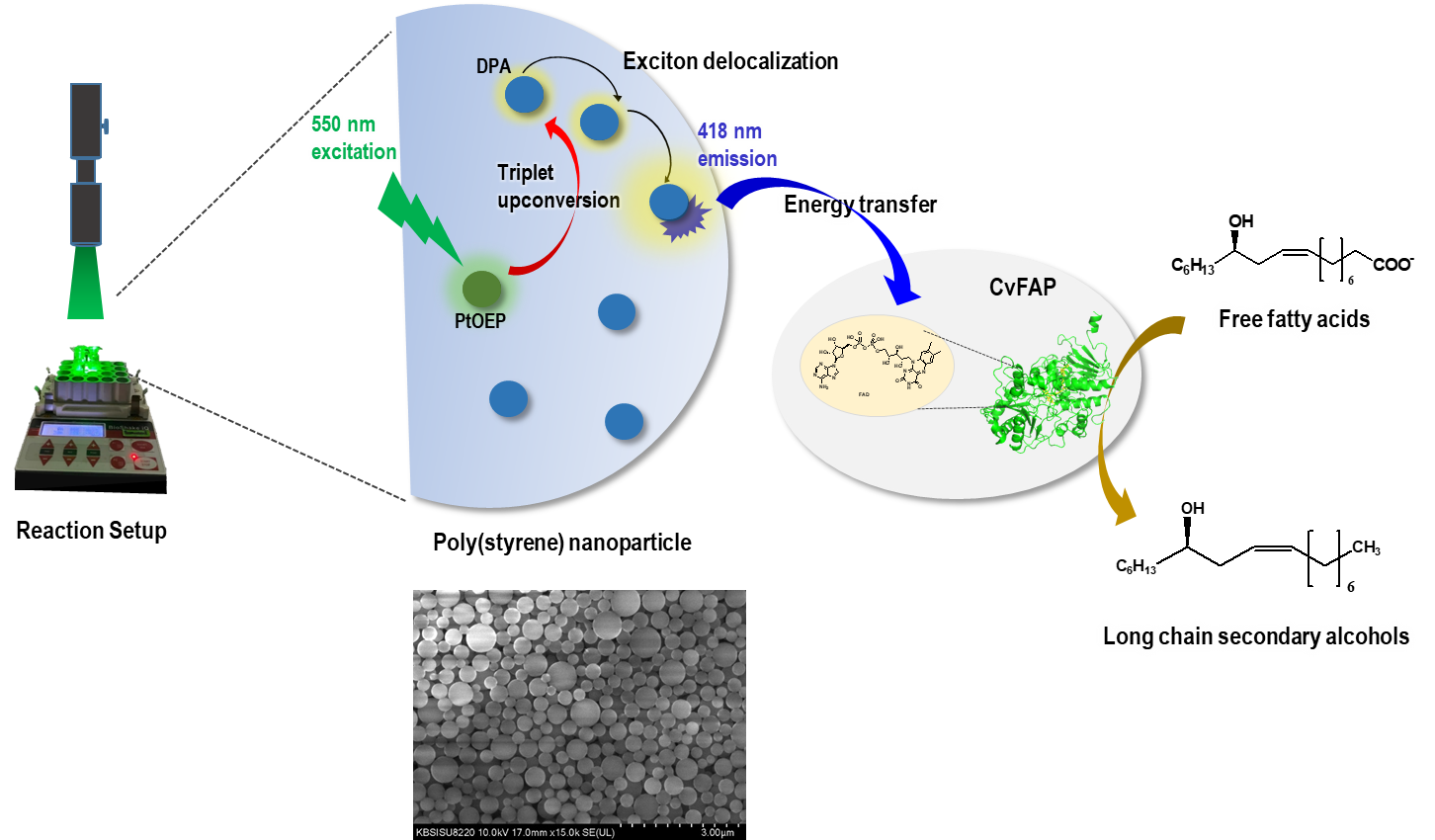


**Figure S13**. Overall concept of the TTA-UC based photoactivated decarboxylase catalysis. Photoexcitation of the ternary PtOEP:DPA@PS nanoparticles with 550 nm light led to upconverted emission of DPA at a wavelength of 418 nm. The TTA-UC emission photoactivates FAD in the flavin-dependent photodecarboxylase (i.e., *Cv*FAP), allowing for the enzymatic decarboxylation of fatty acids into long chain secondary alcohols under green light.

(a)


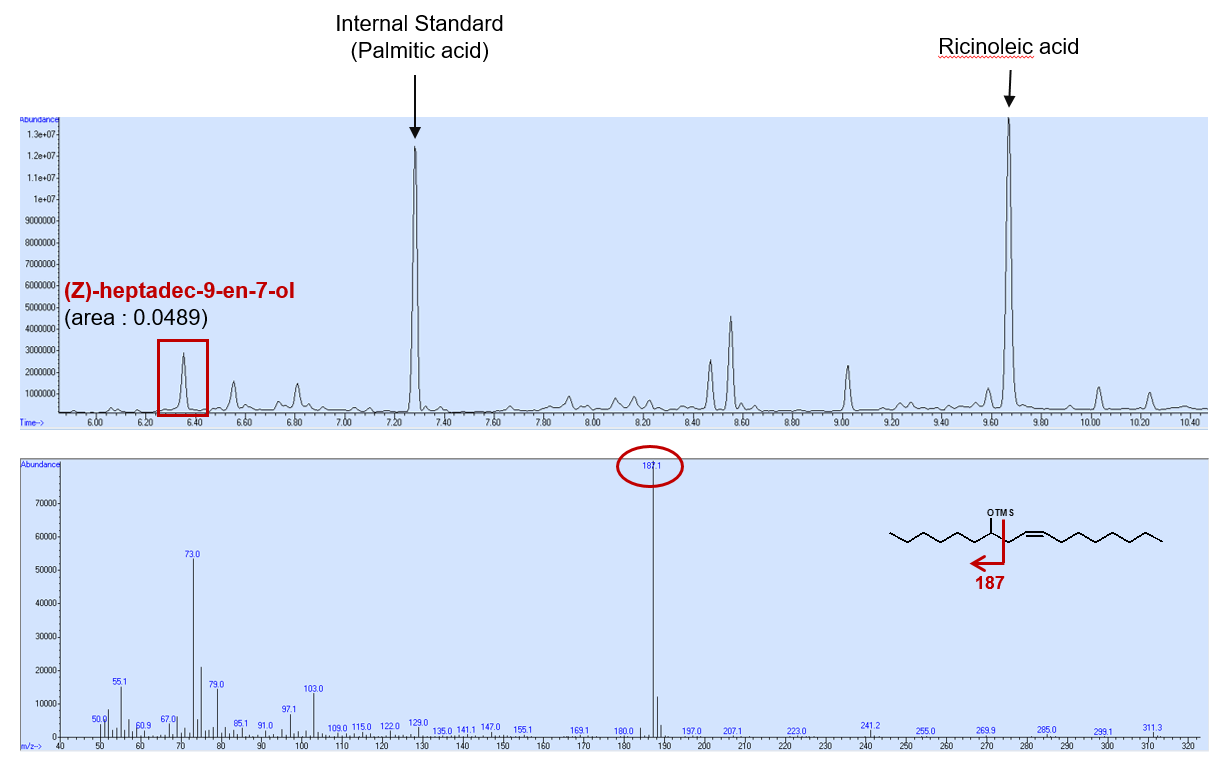


(b)


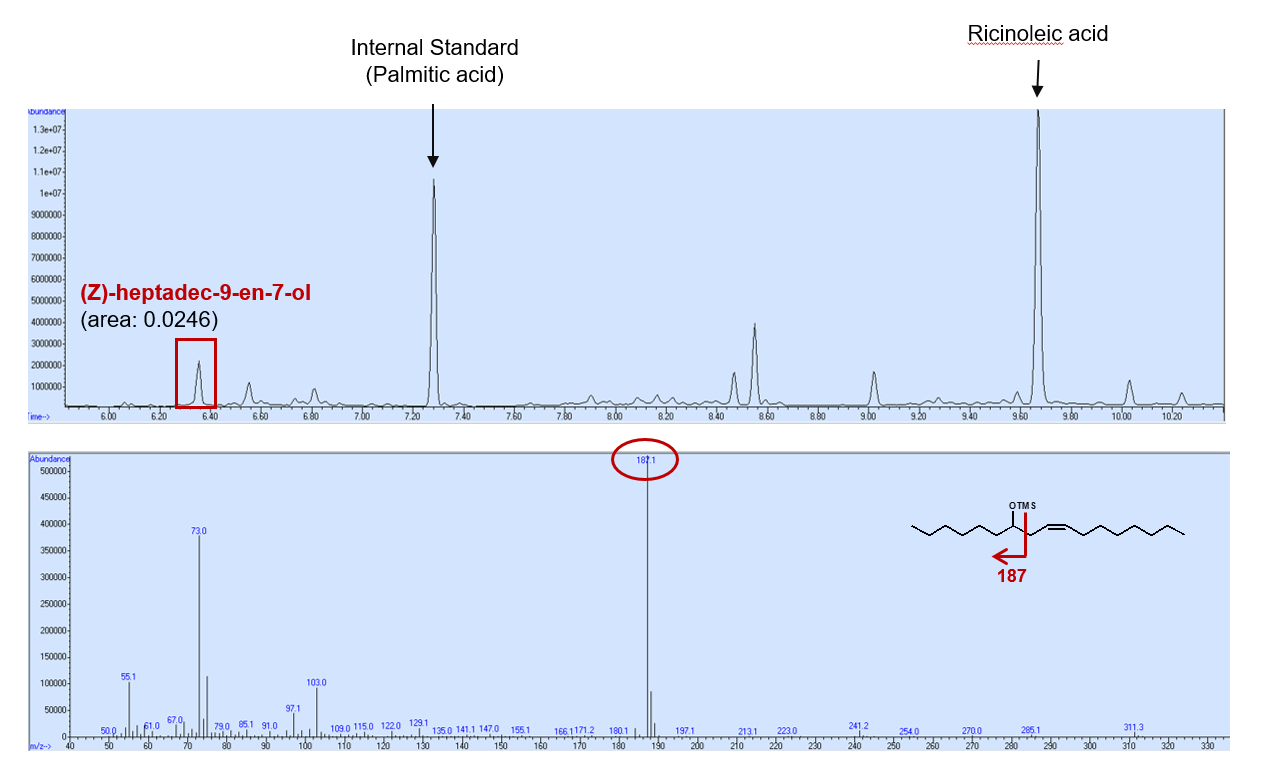


**Figure S14**. GC/MS analysis of the reaction products including (*Z*)-heptadec-9-en-7-ol (**2**) in the presence of PtOEP:DPA@PS nanoparticles (a) and in the absence of PtOEP:DPA@PS nanoparticles (b).

**Figure S15**. Biotransformation of ricinoleic acid (**1**) into (*Z*)-heptadec-9-en-7-ol (**2**). The reaction was carried out by the recombinant *E. coli* cells expressing *Cv*FAP under green light (*λ* = 550 nm) for 420 min. The biotransformations were performed in the buffer, in the buffer containing the polystyrene nanoparticles only, in the buffer containing the DPA@PS nanoparticles, and in the buffer containing the PtOEP:DPA@PS nanoparticles. Reaction conditions: *c*(Ricinoleic acid) = 5 mM, *c*(Cat) = 7.2 g_CDW_ L^-1^, *c*(DPA) = 15 μM for both DPA@PS nanoparticles, and PtOEP:DPA@PS nanoparticles.


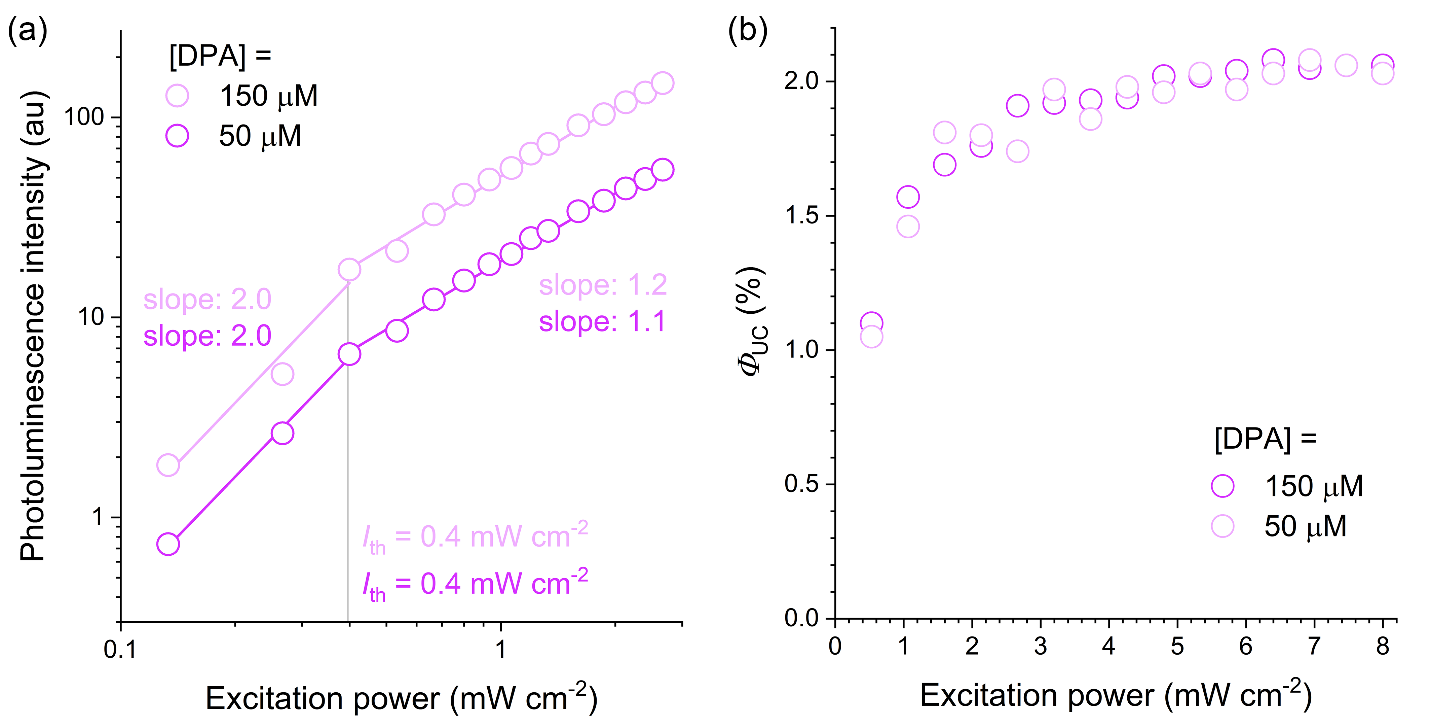


**Figure S16**. (a) Double-logarithmic plots of the photoluminescence intensity of TTA-UC nanoparticle (i.e., PtOEP:DPA@PS nanoparticle) suspension doped with different concentrations of DPA as functions of the photoexcitation power. The threshold photoexcitation power (*I*_th_) is included. (b) The corresponding upconversion fluorescence quantum yields.

**Figure S17**. Decarboxylation of ricinoleic acid (**1**) into (*Z*)-heptadec-9-en-7-ol (**2**) was carried out by the recombinant *E. coli* cells expressing *Cv*FAP under blue light (*λ* = 450 nm). The reaction was performed in the presence of the PtOEP:DPA@PS nanoparticles. Reaction conditions: *c*(Ricinoleic acid) = 5 mM, *c*(Cat) = 7.2 g_CDW_ L^-1^, *c*(DPA) = 15 μM.

**Supplementary References**

1. Huijbers, M. M. E.; Zhang, W.; Tonin, F.; Hollmann, F., Light-Driven Enzymatic Decarboxylation of Fatty Acids. *Angew. Chem. Int. Ed.* **2018,** *57* (41), 13648-13651.

2. Zhang, W.; Ma, M.; Huijbers, M. M. E.; Filonenko, G. A.; Pidko, E. A.; van Schie, M.; de Boer, S.; Burek, B. O.; Bloh, J. Z.; van Berkel, W. J. H.; Smith, W. A.; Hollmann, F., Hydrocarbon Synthesis via Photoenzymatic Decarboxylation of Carboxylic Acids. *J. Am. Chem. Soc.* **2019,** *141* (7), 3116-3120.

3. Seo, E. J.; Kang, C. W.; Woo, J. M.; Jang, S.; Yeon, Y. J.; Jung, G. Y.; Park, J. B., Multi-level engineering of Baeyer-Villiger monooxygenase-based *Escherichia coli* biocatalysts for the production of C9 chemicals from oleic acid. *Metab. Eng.* **2019,** *54*, 137-144.

4. Cha, H. J.; Hwang, S. Y.; Lee, D. S.; Kumar, A. R.; Kwon, Y. U.; Voss, M.; Schuiten, E.; Bornscheuer, U. T.; Hollmann, F.; Oh, D. K.; Park, J. B., Whole-Cell Photoenzymatic Cascades to Synthesize Long-Chain Aliphatic Amines and Esters from Renewable Fatty Acids. *Angew. Chem. Int. Ed.* **2020,** *59* (18), 7024-7028.

5. Zhang, W.; Lee, J. H.; Younes, S. H. H.; Tonin, F.; Hagedoorn, P. L.; Pichler, H.; Baeg, Y.; Park, J. B.; Kourist, R.; Hollmann, F., Photobiocatalytic synthesis of chiral secondary fatty alcohols from renewable unsaturated fatty acids. *Nat. Commun.* **2020,** *11* (1), 2258.

6. Askes, S. H.; Brodie, P.; Bruylants, G.; Bonnet, S., Temperature Dependence of Triplet-Triplet Annihilation Upconversion in Phospholipid Membranes. *J. Phys. Chem. B* **2017,** *121* (4), 780-786.
